# New hospital structure in the twenty-first century: the position of level III (tertiary) neurological and stroke care in a changing healthcare system

**DOI:** 10.1186/s40064-016-3710-3

**Published:** 2016-11-29

**Authors:** Tamás Szentes, László Kovács, Csaba Óváry

**Affiliations:** 1 National Healthcare Service Center, Budapest, Hungary; 2 National Public Health and Medical Officer Service, Budapest, Hungary; 3 Department of Public Health, Faculty of Medicine,, Semmelweis University, Budapest, Hungary; 4 IFUA Horváth & Partner’s, Stuttgart, Germany; 5 National Institute of Clinical Neurosciences, Budapest, Hungary; 6 ÁNTSZ Országos Tisztifőorvosi Hivatal, Albert Flórián út 2, 1097 Budapest, Hungary

**Keywords:** Neurology, Healthcare system, Hospital

## Abstract

**Aim:**

The determination of the necessary capacity and number of neurology wards of level III progressivity that can be defined in the system of criteria detailed in this article and which possess optimal operating conditions in Hungarian terms.

**Methods:**

We used the National Health Insurance Company’s database to calculate case numbers and capacity for different levels of neurological and stroke care. We also revised the allocation of advanced diagnostic and therapeutic technologies, and proposed changes, based on health insurance data. We also discussed these propositions with clinical experts to test their viability.

**Results:**

We determined the adequate number of organisational units capable of providing special neurological healthcare services on the basis of the basic data of the Hungarian healthcare system, specifying this number as 6 instead of the current 11.

**Conclusions:**

In our study, we have identified significant bias in the nationwide level of neurological and stroke care organisation, which needs revised allocation of healthcare resources. Naturally, this can only be carried out through the restructuring of the emergency care system and the expansion of pre-hospital care.

## Background

Thanks to the development of medical science and technology and the changing needs of the population the economic crisis of 2008 would probably enhance structural transformations that started in the healthcare system within all areas. Hospitals as structures are quite resistant to changes (McKee and Healy 2002), but the current change affects especially those hospitals, which operate with advanced technology and trained human resources. Healthcare systems of the former socialist countries of Central Europe where the workforce drain has created a shortage of trained medical human resources, are more vulnerable, therefore much greater attention must be paid to planning in this area.

A decision must be made as to whether in a certain area preference should be given to centralised or decentralised organisation of healthcare, how tasks should be distributed between the various levels and participants of the healthcare system, and at what rate services provided outside institutions should be expanded, etc.

With regard to the operation of neurological wards the objective in the member states of the European Union is the implementation of a unified approach and practice (Struhal et al. 2013; Leone et al. 2013). A tertiary neurology ward of level III progressivity (Johns Hopkins Medicine http://www.hopkinsmedicine.org/patient_care/pay_bill/insurance_footnotes.html) is positioned in the responsive subsystem of the healthcare system, necessarily within a progressive institution that operates across a broad professional spectrum. According to the John Hopkins definition of tertiary service, this is a form of specialised care, that cover a wider area than that served by a local service or trust and include ‘tertiary’ services taking patients from secondary services. How wide an area should be served by such a tertiary service is not defined and will differ for different disorders. This type of care is also distinct from supra-regional services, defined as ‘very specialised services’ that sometimes need to be provided in just one center, and are for very rare conditions with very low national case-load.

## Methods

We wished to determine the necessary capacity and number of neurology wards of level III progressivity which possess optimal operating conditions in Hungarian terms.

We relied on the database of the National Health Insurance Fund (OEP) and the findings of a previous major, nationwide study of the epidemiology of stroke carried out at the turn of the millennium (Óváry et al. 2004). We compared these data with neuroepidemiological data found in international literature (Pringsheim et al. 2014; Chin and Vora 2014; Aminoff et al. 2010) in order to obtain the most accurate estimate. We paid special attention to the position of neurological care within the overall healthcare system and to the separation of stroke and neurological care within neurological care. This latter distinction is justified by the fact that, in Hungary, too, stroke care has been demonstrated to be the most effective when carried out in dedicated stroke units and wards (Óváry et al. 2007).

Since the comprehensive epidemiological study in Hungary (Óváry et al. 2004) was carried out more than 10 years ago, we calculated current case numbers as accurately as possible for cases of stroke, which make up at least 50% of neurological care cases, using financing data from the OEP.

## Results

### Designing neurological inpatient care

From the inpatient database of the OEP we identified the case numbers shown in Table 1 as acute stroke events in recent years, using a method not detailed here.

**Table 1:** Development of the number of acute stroke events between 2004 and 2012 in Hungary *Source* National Health Insurance Fund (OEP) database

Year	Number of cases	Number of patients
2004	26,702	25,248
2005	29,928	28,130
2006	24,403	22,665
2007	30,533	28,263
2008	30,845	28,539
2009	32,746	30,288
2010	29,021	26,925
2011	31,882	29,401
2012	34,575	31,718

The financing body registered 818,870 days of care in Hungarian stroke and neurology wards during 12 months, which represents 6.75% of total national inpatient care, but is one percentage point lower in the capital, at 5.5% (see Table 2).

**Table 2:** Days of care between September 2012 and August 2013

	Neurology wards^a^	Stroke wards	All professions
National total (days)	602,878	215,952	12,123,614

^a^Separately on stroke and neurology wards

In order to design, the capacities with which institutions or organisational units can adequately meet the needs of the population must be determined correctly. Neurology wards operating at level III progressivity must attend to persons with care needs of level III progressivity, as well as cases resulting from the care needs of level II progressivity of a narrower segment of the population and the care needs of level I progressivity of an even narrower segment.

It is very important to state, that regional healthcare obligations must be separated for elective and emergency healthcare needs, because, due to considerations of patient safety, emergency care tasks can only be assigned to a progressive healthcare structure, since the patient selection outside institutions is not yet adequate. This naturally distorts regional healthcare obligations towards progressive points of care by the extent of emergency cases. The outcome of many clinical profiles greatly depends on how quickly patients can receive adequate care (Minnerup et al. 2014), which must also be assisted by services provided within institutions (Nelson et al. 2011).

On the basis of data received, we determined empirically the structure of a neurology ward operating at level III progressivity. We kept in mind that the ward should possess efficient and transparent capacities and considered what level of progressivity partner professions require.

We based the calculation of capacities on case numbers of the given level of progressivity—that is, III, II, and I—and the number of days of care associated with them. However, certain tendencies—the fluctuation of case numbers, the foreseeable development, technological changes, and the redistribution of tasks—which necessitate a correction of the capacities determined on the basis of data, must have also been taken into account. The definition of technological and medical competences must be emphasised, as it has been done in the case of stroke care (Nguyen et al. 2012; Janjua et al. 2012).

At the same time, 62,153 of the 72,699 cases treated in Hungarian neurology and stroke wards were reported by healthcare providers with a diagnosis of stroke (85.5%) (see Table 3). If we take into account that according to the previous national epidemiological study the number of acute stroke events in Hungary could be put realistically at around 36,000/year at the turn of the millennium (Óváry et al. 2004), the 50-50 ratio of stroke and general neurological case numbers generally accepted in international literature is re-established. From the above it follows that when planning national healthcare, neurological care of 36,000 cases each of acute stroke and neurological profiles, with hospital admittance required for chronic post-stroke condition should be prepared for in the future, too (see Table 3).

**Table 3:** Patient data according to BNO^a^ groups from 2012 September to 2013 August. (Neurology inpatient care only)

Patient group	Patients’ number	Cases	Days accounted	Days on ward	Weight	1000 HUF
Stroke and neurology combined	60,413	72,699	498,517	505,156	65,754	9,591,844
Stroke	52,417	62,153	422,674	428,446	58,460	8,493,610
Epilepsy	3,588	3891	22,904	23,092	2741	399,285
Dementia	1654	1765	18,813	18,901	1695	245,439
Inflammatory diseases of the nervous system	271	324	3218	3225	369	54,346
Diseases of the central nervous system	159	221	1561	1846	19	37,932
Diseases of the extrapyramidal system	822	939	7696	7707	548	79,814
Neurodegenerative diseases	394	472	3751	3831	238	34,882
Multiple sclerosis	1588	1879	10,656	10,685	1009	147,494

^a^ *ICD* International Statistical Classification of Diseases

A particularly important finding of the summary according to Diagnosis Related Groups (DRG) is the distribution of costs according to financed DRGs/weighting numbers; within the main neurological diagnostic codes 88.6% of financed care is reported under stroke. Epilepsy, the second most frequent neurological profile in adults accounts for 4.16% of financing, followed by dementia, multiple sclerosis, extrapyramidal diseases, inflammatory diseases of the nervous system, etc.

A particular feature of healthcare in Hungary is that if we examine the diseases cared for according to DRG groups, but disregarding the professional code of the ward attending to the patient, we find a surprising result in terms of financing (Majláth et al. 2013). In contrast to the 9.5 billion HUF of annual financed costs of about 72,000 neurological cases the amount actually paid out for neurological disease codes is nearly double the amount accounted for at neurology and stroke wards at almost 18 billion HUF, while case numbers are 1.77 times higher at nearly 130,000 (see Table 4). 79.2% of financed care, over 14 billion HUF, arises from stroke care.

**Table 4:** Patients and financing according to BNO^a^ groups within 12 months

	Number of patients	Number of cases	Weight	1000 HUF
All neurological diseases	96,889	128,549	123,136	17,917,528
Stroke	75,803	99,177	97,656	14,191,749
Epilepsy	7467	9567	6280	924,353

^a^ *ICD* International Statistical Classification of Diseases

Several evident findings that can be concluded from Tables 3 and 4 would exceed the scope of our present work. In general, however, we can state that 43.4% of cases with neurological profiles are treated outside the current neurological inpatient care system, not in neurology or stroke wards. This fact must be taken into consideration when organising healthcare.

It is also evident that in terms of financing this ratio is even higher, as the more favourably financed neurological cases are more likely to be handled under other professional codes (see Tables 3, 4).

### Suggestions for changing advanced neurological services

In light of our previously presented premises, according to which the care of neurological profiles and stroke patients should take place in special wards appropriate for the disease, we consider that the figure of 800,000 days of care/year would be realistic for all progressivity levels combined, after correction for the anomalies that can be detected in part in the codes. Within this figure the estimated 1% share of cases justifiably requiring the highest level of progressivity, level III gives a probable/estimated figure of 8000 days, which could be met by approx. 6 centres of level III progressivity in addition to their care obligations of lower, level II and I progressivity, if the conditions detailed in Table 5 were fulfilled (for the purposes of comparison the table also contains the recommended system of criteria for healthcare providers of level II progressivity). In this way, each of these centres can cover a population of about 1.3 million on the tertiary level. These centres have also other responsibilities besides tertiary level care; they need to cover catchment areas on II and I level as well; however for smaller populations, since the number of health care providers is higher on lower levels. Even those who provide tertiary level care should cover level I for a population of 100–150,000.

**Table 5:** Criteria of the planned neurological care of level II and III progressivity by hospital organisational, architectural, medical technological as well as human resource system level

		Neurology II	Neurology III
Inpatient and chronic care	Required healthcare area (×1000 patients)	150–300	1800
	Professional environment (partner professions required for care)	Intensive therapy unit, radiology, central laboratory	Intensive therapy unit, (neuro)radiology, neuro-intervention., otolaryngology, ophthalmology, central laboratory, neurosurgery
	Presence of the profession in the institution (independent area/background consultation)	Independent area	Independent area
	Minimum number of beds required	20–30	60–70
	Minimum number of physicians required (per 10 beds, broken down by specialists and residents)	3 Specialists, 2 general physicians	6 Specialists 2 general physicians, with the competences listed in Table 6
	Level of medical competence (proficiency, according to the professions detailed)	Neurology	Neurology, stroke-licence, neuroradiology, neuro-inervention, neurosurgery
	Minimum number of nurses required (per 10 beds, broken down by specialist and assistant nurses)	4 specialist nurses 2 assistant nurses	4 specialist nurses, 2 assistant nurses
	Other professional staff (per 10 beds, indicating the profession)	Physiotherapist/20 beds	Physiotherapist/10 beds Neuroradiological professional assistant
	Competence level of professional staff (proficiency, by profession)	–	
	Technical requirements (operating theatre, gym, etc.)	Gym	Gym
	Diagnostic requirements (at the level of the institution)	CT	MR, CT,DSA, X-ray, neurointervention
	Diagnostic requirements (at the level of the profession)	Ultrasound, microscope	Neurosonology (Doppler ultrasound, TCD, soft tissue ultrasound) neurophysiology, neuropsychology
	Special requirements for the building (e.g. heliport, optimal volume requirement)		Heliport
	Duty service (continuous, rotation)	Continuous	Continuous
	Comments	The presence of basic professions must be ensured at consultation level if the institution lacks them (cardiology, surgery, urology, gynaecology, psychiatry, paediatrics)	The presence of basic professions must be ensured at consultation level if the institution lacks them (cardiology, surgery, urology, gynaecology, psychiatry, paediatrics)
Specialist outpatient unit	Minimum consulting hours of physicians (consulting hours in a daily breakdown)	24 h/week	266 h/week, according to the breakdown shown in Table 7
	Level of medical competence (proficiency, according to professions)		
	Minimum number of nurses required (broken down by specialist and assistant nurses)	1 Specialist nurse	2 Specialist nurses
	Other professional staff	1 Administrator	3 Administrators
	Technical requirements (operating theatre, gym, etc.)	Gym	Gym
	Diagnostic requirements (at the level of the profession)	Access to inpatient diagnostic level	Access to inpatient diagnostic level
	Is the presence of the profession compulsory or optional in the given institution?	Compulsory	Compulsory
	Minimum number of hours required	8 h/day	8 h/day
	Availability (broken down by day parts and days)	5 Full working days, during working hours	5 Full working days, during working hours

In connection with this, Table 6 presents proposed medical competences for level III progressivity, while Table 7 details the hours of inpatient care.

**Table 6:** Details of the competence and capacity levels of medical human resources for care of level III progressivity

Professional competences	Minimum staff numbers
General physician	2
Neurologist	6
Psychiatrist^a^	1
Rehabilitation specialist^a^	1
Clinical neurophysiologist^a^	1
Clinical pharmacologist^a^	1
Neurosonologist^b^	2
Neurosurgeon^a^	1

^a^Also together with other professional qualifications

^b^One of them may be a neurosonological professional assistant

**Table 7:** Details of the hours of outpatient care of competence level III by activity and specialist/non-specialist hours

	Specialist hours	Non-specialist hours	Sum
Stroke and general neurology	80	10	90
Epilepsy	30	30	60
Headaches	16	20	36
Neurophysiology	30	0	30
Otoneurology	10	0	10
Neurosonology	20	20	40
TIA emergency care	20	20	40
Total	206	100	306

*TIA* transient ischaemic attack

## Discussion

In Hungary, the last reform that involved the healthcare system started after the change of government in 2010. The healthcare administration laid down the theoretical framework of this transformation in the program entitled Semmelweis Plan (SP) (http://www.eti.hu/eti/fooldal/4183). The Semmelweis Plan provided a recommendation for the rationalisation and optimisation of the healthcare system, taking into account existing characteristics and needs. It qualified the existing healthcare system as fragmented and pointed out the lack of coordination between institutions, as well as the lack of organisation leading in turn to a lack of integrity in the healthcare system. The SP also indicated the theoretical framework of transformation at macro level and described the methodology of its carrying out, with the main elements as below:

- Situation report, recording initial conditions: capacity, performance, HR, infrastructure, etc. by institutions.
- Defining progressivity levels by professional areas.
- Defining organisational areas (regions).
- Assigning progressivity levels to the professional areas represented in the institution, followed by setting up the institutional hierarchy.
- Setting the correct ratio of capacities between progressivity levels.
- Setting the correct ratio of capacities at regional level, by professions (Kövi and Tóth 2011).

The authors agree with laying down a correct theoretical framework, however, the methodology indicated in the Semmelweis Plan has not yet been broken down to the various levels of the healthcare system; therefore no actual system-level transformation has taken place that would have meaningfully affected the structure and operation of institutions (http://www.korhazszovetseg.hu/archivum/turelmi_idot_kaptak_az_egeszsegugyi_szolgaltatok).

In our study we focus on the hospital segment of the healthcare system, which can provide technology-intensive/high-tech, efficient, and specialised services, and present the methodology of redefining organisational units. In the long run these special units will be linked to a specific institution, as services provided outside institutions at a lower level of progressivity are already available at a relatively high level of safety (Dubois et al. 2006; Black and Gruen 2005). In order to ensure functionality at system level, it has become necessary to determine and precisely define organisational units first within each profession (Tinsley et al. 2010), then in relation to the various partner professions, to individual institutions, and to every element of the healthcare system. With the multidisciplinary approach gaining ground, the role and position of the various professions in the healthcare system and their various levels are being redefined (Boerma 2006).

In our study, assessing the real needs for neurological care, we tried to define locally, in Hungary, the amount of neurological disorders, treated inadequately in other inpatient wards. Although this problem is not different in Western-Europe, our rate of 43.4% for inadequate care is very high. The importance of this rate comes from the fact, that our planned capacities should cover these cases as well.

The organisational units defined in this manner will be the building blocks of individual healthcare institutions, which together comprise the entire healthcare system.

During the planning processes, we must lay down certain premises that define the limits of planning beyond the rules related to medical science. These are system regulation factors (http://www.eti.hu/eti/fooldal/4183) that can be deduced from the interpretation of healthcare as a public duty (Kövi and Tóth 2011), the operation of the healthcare services market (http://www.korhazszovetseg.hu/archivum/turelmi_idot_kaptak_az_egeszsegugyi_szolgaltatok), and the rules for the operation of this market with regard to the given healthcare system.

### Hospital structure in the twenty-first century

On the basis of the aspects of needs defined partly by efficiency, and partly by professional development, we recommend the centralised method. This is substantiated by the fact that the efficiency of healthcare systems that have been set up according to different principles, and are therefore organised differently, naturally varies, where time and regional aspects might have major effects. The Canadian healthcare system, e.g. has been developing in a centralised manner since the 1950s, which has led to an improvement in the efficiency of individual professions, including neurology, since the structure of the healthcare system has ensured a better allocation of resources (Feasby 2006).

The concentration of hospitals is taking or will take place, partly in a spontaneous, and partly in a regulated manner. As a result the number of hospitals is decreasing in all the healthcare systems of Europe, amounting to a drop of 7% between 2000 and 2010 (Hospitals in Europe: Healthcare data 2012). Compared to the necessary extent there is already a scarcity of resources as to the financing of operations, human resources (Rechel et al. 2006), or expensive technology; and this is likely to intensify in the future. It is in part due to the fact that technology-intensive services are gradually being concentrated in a progressively decreasing number of institutions (Wagenaar 2006). An example of this technological concentration to a specific institution, as well as within an institution is the “Core Hospital”, which won an award in the Dutch contest announced for the hospitals of the future, and the popularity of which has been increasing worldwide ever since. The Core Hospital offers a more concentrated organisation of hi-tech services than ever before (Netherlands Board for Health Care Institutions 2005a). In parallel, several services have become available outside hospitals, as well, through on-site tests and mobile radiology. With the spread of telemedicine these services have made inroads into outpatient centres and basic healthcare centres, as well (Black and Gruen 2005). Thanks to this, hospitalisation rates have decreased for several clinical profiles for which they were traditionally high (Marrie et al. 2014), and the time of hospital stays could also be reduced through adequate pre-hospital emergency care (Saifee et al. 2013; Weber et al. 2013). These two parallel processes define the redistribution of tasks. In consequence of these, hospitals will become institutions with increased capacities, capable of providing specific services that require a higher level of technology, and will accordingly include a greater number of professions, while their number will continue to decrease.

For this reason, the structure of hospitals and their organisational units can only be defined if basic principles are laid down:

- The nature and amount of required services must be adjusted to the needs of the population, and when defining the framework for planning foreseeable trends and increasing demand for services must be taken into account (Mathers and Loncar 2006).
- When planning the healthcare system financing limitations, the current volume of resources and expected future expenditure must be taken into account.
- The present state of medical science, available technology, and future trends, which are as yet difficult to predict (genetics, molecular biology, telemedicine, nanotechnology, personalised therapy, autodiagnostics, etc.) have to be taken into consideration (McKee and Healy 2002).
- The utilisation of expensive, specific technologies must be optimised.
- Due to considerations of patient safety, emergency treatment should only be offered in institutions capable of providing care of a high level of progressivity.
- Attention must be paid to harmonisation between partner professions, which means that healthcare units of the same level of progressivity or whose operation can be harmonised with each other, and which possess given technological requirements should be linked within institutions.
- Under the progressivity level of a given organisational unit we mean the highest level of progressivity of its operation, beneath which it provides care at all levels of progressivity.
- The service area of an organisational unit can be largest at its highest level of progressivity, and somewhat smaller at lower levels of progressivity.
- Any population must be granted access to healthcare at all levels of progressivity through healthcare providers.

### The position of an organisational unit within an institution

As a result of the high specialisation within the professions the need raises to set up autonomous and independent organisational units that integrate all subspecialities of the given profession. However, in the terms of intra- and inter-professional relations, we are convinced that the integration of various professions is the necessary direction for future development, since specialised areas are becoming increasingly dependent on other professions (Tinsley et al. 2010). A constant connection must be kept with the supportive professional areas in the interest of efficient healthcare (van Nooten et al. 2015; Demartin et al. 2014). Moreover, the presence of and the consultative support between partner professions also improve the functioning of the entire institution (Ali et al. 2010).

Another determining factor is the *distribution of tasks* between organisational units within the profession. This means that when defining the resources—technology, human resources—and capacities of a given organisational unit we must take into account what is expected of it and how it should relate within the profession to other healthcare providers. For well-defined tasks, such as stroke care, this regulation is already in place. The Polish example provides a good description of the rules for this type of differentiation within healthcare units—there exist comprehensive stroke units, primary stroke units and neurology wards which are not capable of providing stroke care. These organisational units possess well-defined healthcare competences (Sarzysnka-Dlugosz et al. 2013), which significantly improves the access to efficient special services (Di Carlo et al. 2011). This task is considerably more difficult in the case of a complex service structure, for example, in neurology. Accordingly, the presence of special diagnostic and therapeutic modalities is indispensable in an institution that provides advanced, tertiary neurological care, and the number thereof is constantly increasing to meet this need (Schapira 2014; Schapira 2013; Schapira and Hillbom 2012). The correct implementation of effective, proven procedures improves efficiency at system level, as well (Dirks et al. 2012).

“Time is brain” and this is also a major concern, at least in stroke care. We do not think that all catheter thrombectomy centers should also provide complete level 3 neurology care. However, all level 3 centers must provide thrombectomy. The difference between these statements comes from the fact, that estimated numbers of potentially treatable big vessel occlusion strokes are higher, than the capacity and the availability of level 3 centers, while increasing the number of level 3 centers would inadequately allocate our resources.

The biggest issue in deciding between secondary or tertiary stroke care is the triage before hospital admission, and how we can select those patients, who are eligible for thrombectomy (English et al. 2016). Recently, there’s no widely accepted method for that, and some patients primarily admitted to a secondary level neurology ward must be transferred to a tertiary ward, *or* a thrombectomy center, after the initial brain CT or CT angiography.

The *centralisation* of hospital care requires that institutions with special facilities possess greater capacity and treat greater number of, and more severe cases, and also affects the structure of organisational units and their necessary resources (Wagenaar 2006; University Medical Centre Groningen 2005), which means that more resources must be provided to them.

This trend is partly confirmed by the weakening of the institutional orientation of healthcare, which moves less specific services outside of hospitals by improving them (Clemens et al. 2014; Ten 2012; Martinez-Alcala et al. 2013).

When designing an institution and organisational unit we must also take into consideration several factors the effects of which can only be manifested on the medium run. These factors include a reduction in the number of preventable diseases, more affordable forms of healthcare, together with the creation of the structures that provide them and their implementation at system level. The aspects of the training of human resources and those of the health education of the population are indispensable for the future optimisation of the structure.

## Summary

In our present work we concluded that six neurology wards operating at tertiary level can carry out the required amount of healthcare services of a suitable quality in Hungary, with the necessary resources being at disposal. This ensures the desired allocation of resources, which are necessary to provide services of a suitable quality and satisfy the criteria of patient safety. Access to services within an appropriate timeframe can be improved through the support of pre-hospital services, either diagnostic (Handschu et al. 2014) or curative (Saifee et al. 2013; Weber et al. 2013; Audebert et al. 2013).

 In contrast to our findings, at present 11 neurology wards operating at Level III progressivity exist in Hungary, with a capacity of 393 beds. In our view the maintaining of this situation already results in a shortage of the suitable resources for organisational units, which are in consequence unable to provide the required services in an appropriate quality. In the healthcare system only structures with efficient services can be justifiably maintained, as those operating at a low level of efficiency generate further costs in the healthcare system (Chevreul et al. 2013). The incorrect utilisation of direct healthcare expenditure can considerably increase indirect expenses (Olesen et al. 2012) which exacerbate inadequate patient care, particularly at low progressivity level, creating a patient drain in outpatient care. The relative shortage of resources will become more intense in the future, and maintaining the status quo will be detrimental to aspects of patient safety.

The sectoral trends outlined above reveal that a centralisation of services with a high technological requirement will necessarily take place. This process, however, should not be spontaneous, but should take into account professional requirements, as well as the needs of partner professions, in line with the aspects the importance of which will continue to increase with specialisation. It is exactly for these reasons that we consider it important that the profession should position itself in the future taking into account not only its internal trends of professional development, but also the determinative factors that stem from the necessary transformation of the healthcare system that defines its own operating environment.
